# Clinicopathological analysis of diffuse large B-cell lymphoma using molecular biomarkers: a retrospective analysis from 7 Hungarian centers

**DOI:** 10.3389/fonc.2023.1224733

**Published:** 2023-09-08

**Authors:** Anett Balikó, Zsolt Szakács, Béla Kajtár, Zsombor Ritter, Attila Gyenesei, Nelli Farkas, László Kereskai, István Vályi-Nagy, Hussain Alizadeh, László Pajor

**Affiliations:** ^1^ Tolna County Balassa János Hospital, Szekszárd, ;Hungary; ^2^ PhD Doctoral School – Interdisciplinary Medical Sciences (D93), Medical School, University of Pécs, Pécs, ;Hungary; ^3^ First Department of Medicine, Medical School, University of Pécs, Pécs, ;Hungary; ^4^ Department of Pathology, Medical School, University of Pécs, Pécs, ;Hungary; ^5^ Department of Medical Imaging, Medical School, University of Pécs, Pécs, ;Hungary; ^6^ Szentágothai Research Centre, University of Pécs, Pécs, ;Hungary; ^7^ Institute of Bioanalysis, Medical School, University of Pécs, Pécs, ;Hungary; ^8^ South-Pest Hospital Centre – National Institute for Infectology and Haematology, Budapest, ;Hungary

**Keywords:** diffuse large B-cell lymphoma, MYC, BCL6, BCL2, IHC, FISH, prognosis

## Abstract

**Background:**

The clinical and genetic heterogeneity of diffuse large B-cell lymphoma (DLBCL) presents distinct challenges in predicting response to therapy and overall prognosis. The main objective of this study was to assess the application of the immunohistochemistry- and interphase fluorescence *in situ* hybridization (FISH)-based molecular markers in the diagnosis of DLBCL and its prognostic value in patients treated with rituximab-based immunochemotherapy.

**Methods:**

This is a multicenter, retrospective study, which analyzed data from 7 Hungarian hematology centers. Eligible patients were adults, had a histologically confirmed diagnosis of DLBCL, were treated with rituximab-based immunochemotherapy in the first line, and had available clinicopathological data including International Prognostic Index (IPI). On the specimens, immunohistochemistry and FISH methods were performed. Germinal center B-cell like (GCB) and non-GCB subtypes were classified by the Hans algorithm. Outcomes included overall survival (OS), event-free survival (EFS), and EFS at 2 years (EFS24). For survival analysis, we used Kaplan-Meier curves with the log-rank test and multivariate Cox regression.

**Results:**

A total of 247 DLBCL cases were included. Cases were positive for MYC, BCL2, BCL6, and MUM1 expression in 52.1%, 66.2%, 72.6%, and 77.8%, respectively. *BCL6* translocation, *BCL2* gene copy number (GCN) gain, *IGH::MYC* translocation, *MYC* GCN gain, *IGH::BCL2* translocation, and *BCL6* GCN gain were detected in 21.4%, 14.1%, 7.3%, 1.8%, 7.3%, and 0.9%, respectively. At a median follow-up of 52 months, 140 patients (56.7%) had disease progression or relapse. The Kaplan-Meier estimate for EFS24 was 56.2% (CI: 50.4–62.8%). In univariate analysis, only IPI and BCL6 expression were significant predictors of both OS and EFS, whereas MUM1 predicted EFS only. In multivariate analysis, the IPI score was a significant independent negative, whereas MIB-1 and BCL6 protein expressions were significant independent positive predictors of both OS and EFS.

**Conclusion:**

In our study, we found that only IPI, BCL6 protein expression and MIB-1 protein expression are independent predictors of survival outcomes in DLBCL. We did not find any difference in survival by GCB vs. non-GCB subtypes. These findings may improve prognostication in DLBCL and can contribute to designing further research in the area.

## 1 Introduction

Diffuse large B-cell lymphoma (DLBCL) is the most common high-grade non-Hodgkin lymphoma accounting for 30–40% of B-cell non-Hodgkin lymphomas (1). DLBCL has considerable biologic, molecular, and clinical heterogeneity resulting in different responses to therapy (2). DLBCL is a potentially curable disease with an overall 60–70% chance of achieving durable complete remission (CR) with the currently used standard first-line immunochemotherapy. However, 30–40% of patients are either refractory to first-line treatment or experience relapse and eventually will die of disease progression (3). Patients achieving remission lasting for 24 months or more from the end of first-line treatment have favorable prognosis at long term (4–6). The diagnosis and subtyping of DLBCL have come far to date, from morphological assessment of tissue slide to now, where numerous ancillary tests are a prerequisite, including immunophenotyping performed by immunohistochemistry (IHC), cytogenetics, and detailed molecular testing to classify the disease based on cell of origin (COO). With the advent of novel therapeutic options, it is expected that molecular subtyping of DLBCL at diagnosis will allow prognostic stratification of patients into distinct subgroups which could provide preclinical rationale for therapeutic targeting the involved pathways and paving the application of personalized treatment. The original DLBCL molecular classification using DNA microarray-based technology was initially described by Alizadeh et al. (7). This technology allowed the analysis of transcriptional gene expression pattern, and distinguished two major COO subtypes of DLBCL: germinal center B-cell like (GCB) and activated B-cell like (ABC). This resulted in the molecular subtyping of 80–85% of all DLBCL cases. Treated with standard immunochemotherapy, the ABC subgroup had poorer 5-year survival compared to the GCB group (16% vs. 76%, respectively).

Hans (8), Choi (9), and Tally (10) are the most established algorithms that are based on IHC to assess COO with the Hans algorithm being the most commonly used in clinical practice. However, the clinical significance of COO subtyping using IHC remains controversial, and the assessment of COO by gene expression profiling rather than IHC is better associated with the prognosis of DLBCL (11).

Translocations, gene rearrangements, and protein expression patterns including but not limited to BCL6, BCL2, and MYC have been shown to have prognostic implications.

The human proto-oncogene *BCL6* was identified from chromosomal breaks at 3q27, is expressed predominantly in germinal center B-cells (12, 13), and functions as a sequence-specific transcriptional repressor (14, 15). Mutation or translocation of *BCL6* gene has been implicated in the pathogenesis of certain subtypes of DLBCL (12). *BCL6* is frequently affected by chromosomal translocations, occurring more frequently in ABC DLBCL. There is no consensus on the effect of *BCL6* translocation on prognosis of DLBCL, especially in the rituximab era, with studies showing either favorable (16, 17), neutral (18, 19), or unfavorable outcomes (20).

The BCL2 protein is a central regulator of the mitochondrial apoptotic pathway and its gene is located on the chromosome arm 18q21 (21). The primary function of *BCL2* is to promote cell survival by inhibiting apoptosis (22–24). The *BCL2* gene is overexpressed in many cancers including DLBCL and is usually associated with drug resistance (25). In DLBCL, the ratio of BCL2-positive cases is highly variable, ranging from 20% to 80% across studies that used IHC (26, 27). Some studies showed BCL2 protein expression as a marker of poor prognosis in GCB subtype of DLBCL (26, 28), while others reported it to be a marker of poor prognosis in ABC-DLBCL, only (29).

Chromosomal translocations dysregulating *MYC* (8q24) were reported in 5–15% of GCB DLBCL cases (17), while MYC protein detection in 5–40% of DLBCL cases (30–32). *MYC* gene translocation was associated with a very poor outcome in DLBCL (33–36). *MYC* translocation in DLBCL is usually associated with other gene abnormalities, e.g., *BCL2* or *BCL6* rearrangements (37, 38). Some studies show that about 30% of DLBCL co-express high levels of MYC and BCL2 proteins, which cases are called ‘double expressor’ DLBCL (DEL) (39).

Although substantial knowledge has accumulated about prognostication in DLBCL, a lot of questions have remained unanswered. The main objective of this study was to describe the distribution of and to test the prognostic ability of IHC- and interphase fluorescence *in situ* hybridization (FISH)-based molecular markers in a Hungarian cohort of newly diagnosed DLBCL patients from the rituximab era.

## 2 Patients and methods

This multicenter, retrospective study was approved by the Committee of Science and Research Ethics (ETT-TUKEB) under reference number 50268-8/2017.

### 2.1 Study population and sample assessment

This study is reported according to the STROBE Statement (40).

A total of 342 adult patients diagnosed with DLBCL were assessed. Tissue samples were sent by 7 Hungarian Hematology centers to the central hematopathology lab in the Department of Pathology, Medical School, Clinical Centre, University of Pécs between Jan 2010 and Mar 2017. All samples were reviewed by 2 senior pathologists. The final pathology report was established according to the diagnostic criteria of the 2016 WHO Classification of Tumours of Haematopoietic and Lymphoid Tissues (37). Out of the 342, 95 cases were excluded due to incomplete clinicopathological data, so a total of 247 cases were further assessed in the study. All specimens were fixed in 10% neutral formalin and conventional paraffin embedding was performed.

IHC was carried out according to standard protocols using CD10, clone 56C6; CD20, clone L26 (Visionbiosystems Novocastra, UK); MUM1/IRF4, clone MUM1p; BCL2, clone 124; BCL6, clone PG-B6p (Dako, Denmark); MYC, clone Y69 (Abcam, UK); Ki-67, clone B56 (Hisztopatológia Kft., Hungary) specific primary antibodies as well as Envision+ System-HRP (DakoCytomation, Denmark) and Bond Polymer Refine Detection (Leica Biosystems, UK) developing reagents. According to the Hans algorithm, at least 30% reactivity–either membranous or nuclear–is required for a tumor to be considered positive for a particular marker. The tumors were classified as GCB-like when exhibiting CD10+ (BCL6+/-, MUM1+/-) or CD10-, BCL6+, MUM1- phenotype. Non-GCB/ABC like was the subtype in the case of all the other–CD10-, BCL6+, MUM1+ or CD10-, BCL6-, MUM1+–phenotypic variables (8). DEL was defined as combined BCL2 (≥50%) and MYC (≥40%) positivity (37).

FISH was performed using 5 μm paraffin tissue sections for *IGH::MYC, IGH::BCL2, BCL6* rearrangement and for *MYC, BCL2, BCL6* gene copy number (GCN) gain. For these, Vysis IGH/MYC/CEP8 TC-DF, Vysis LSI IGH/BCL2 DC-DF, and Vysis LSI BCL6 (ABR) DC Break Apart probes (Abbott Molecular Inc., USA) were used. FISH reactions were analyzed in Zeiss Axioplan-MOT II fluorescent microscope and evaluated by means of ‘grid sampling’ and ‘color rationing’ methods (41). We have used double fusion FISH probes to detect *IGH::BCL2* and *IGH::MYC* fusions, since non-*IG BCL2* fusions are rare in DLBCL, and the prognostic significance of non-*IG MYC* fusions is controversial. Cases with non-*IG BCL2* or *MYC* fusions showed signal patterns indicating *BCL2* or *MYC* gain in our series. A tumor was defined positive for rearrangement using IGH/MYC, IGH/BCL2, and BCL6 probes if the fusion or the dissociated FISH signs occurred in at least 50% of the nuclei. The definition of *MYC, BCL2*, and *BCL6* GCN gains included the lack of the polyploidy of the relevant chromosome and/or detection of at least one extra gene copy at least in 50% of the nuclei or identifying ‘double minutes’ and/or ‘beaded lace-like’ signals and/or uncountable (homogeneous staining region or cloudy-like) signals.

### 2.2 Clinical assessment

Detailed clinical and laboratory data including treatment regimen and clinical outcomes (overall survival, OS; event-free survival, EFS; EFS at 24 months, EFS24) were collected from patients’ records, then, all data were reviewed by a senior hematologist. The clinical stage was evaluated by the modified Ann Arbor and Lugano classifications (42, 43). CR, partial response, progression, refractory disease, and relapse were defined according to the International Working Group response criteria for lymphoma (44). EFS was defined as the time from the end of first line treatment until the earliest occurrence of disease progression or death of any cause. EFS24 was defined as being alive and free of any disease related event 24 months from the end of therapy.

### 2.3 Statistical analysis

In univariate statistics, Chi^2^ test was used to analyze the association across clinical variables. Kaplan-Meier curves with a median estimate (with 95% confidence interval, CI) and the log-rank test were used for univariate survival analysis. Multivariate Cox regression analysis was applied to identify independent prognostic factors for the outcomes (OS and EFS). The models were adjusted for gender, international prognostic index (IPI) subgroups, IHC (CD10, BCL6, MUM1, high MIB-1 >90%, MYC, and BCL2) and for FISH findings (*BCL6* translocation and *BCL2* GCN gain). In general, *P <*0.05 value was considered statistically significant. Statistical analysis was performed using R statistical software version 4.2.0 (45) and the survminer package v0.4.9 statistical software (46).

## 3 Results

**Table 1** summarizes the clinical characteristics of the 247 patients included. The median age at the time of diagnosis was 65 years (range: 19–91 years), 65.2% of patients were >60 years, 46.6% were male, 42.9% had an elevated serum lactate dehydrogenase and 74.1% had an advanced (stage III/IV) disease. The majority (94.7%) were treated with R-CHOP or similar regimens, the most common salvage therapies were the R-ICE and R-DHAP protocols.

**Table 1:** Characteristics of patients included.

Total no.	n	%
Age (years)		
≤ 60	86	35
> 60	161	65
Sex		
Male	115	47
Female	132	53
IPI		
0–1	58	24
2	69	28
3	85	34
4–5	35	14
Presentation		
Nodal	185	75
Extranodal	62	25
Stage		
I-II	64	26
III-IV	183	74
ECOG PS		
0–1	133	54
2–4	114	46
LDH		
Normal	141	57
Elevated	106	43

IPI, International Prognostic Index; ECOG PS, Eastern Cooperative Oncology Group Performance Status; LDH, lactate dehydrogenase.

### 3.1 IHC and FISH distribution, and their associations

**Table 2** summarizes the IHC findings, **Table 3** shows FISH distribution by COO subtypes. A total of 234 and 220 patients had available IHC and FISH data, respectively.

**Table 2:** Immunohistochemical staining results by cell of origin subtypes.

	GC, n (% of total)	NGC, n (% of total)	GC + NGC, n (% of total)
MYC			
Negative	40 (47)	72 (48)	112 (48)
Positive	45 (53)	77 (52)	122 (52)
BCL6			
Negative	6 (7)	58 (39)	64 (27)
Positive	79 (93)	91 (61)	170 (73)
MUM1			
Negative	50 (59)	2 (1)	52 (22)
Positive	35 (41)	147 (99)	182 (78)
BCL2			
Negative	38 (45)	41 (28)	79 (34)
Positive	47 (55)	108 (72)	155 (66)
CD10			
Negative	19 (22)	149 (100)	168 (72)
Positive	66 (78)	0	66 (28)
DEL			
Negative	62 (73)	94 (63)	156 (67)
Positive	23 (27)	55 (37)	78 (33)

DEL, double expressor lymphoma; GC, germinal center B-cell like; NGC, non-germinal center B-cell like.

**Table 3:** Interphase fluorescent *in situ* hybridization results by cell of origin subtypes.

	GC, n (% of total)	NGC, n (% of total)	GC + NGC, n (% of total)
*IGH::BCL2* translocation			
Negative	64 (80)	140 (100)	204 (93)
Positive	16 (20)	0	16 (7)
*BCL2* GCN gain			
Negative	73 (91)	116 (83)	189 (86)
Positive	7 (9)	24 (17)	31 (14)
*IGH::MYC* translocation			
Negative	71 (89)	133 (95)	204 (93)
Positive	9 (11)	7 (5)	16 (7)
*MYC* GCN gain			
Negative	78 (98)	138 (99)	216 (98)
Positive	2 (2)	2 (1)	4 (2)
*BCL6* translocation			
Negative	71 (89)	102 (73)	173 (79)
Positive	9 (11)	38 (27)	47 (21)
*BCL6* GCN gain			
Negative	80 (100)	138 (99)	218 (99)
Positive	0	2 (1)	2 (1)
DHL			
Negative	76 (95)	140 (100)	216 (98)
Positive	4 (5)	0	4 (2)

GC, germinal center B-cell like; NGC, non-germinal center B-cell like; GCN, gene copy number; DHL, double hit lymphoma.

A positive IHC staining for MYC, BCL2, BCL6, and MUM1 protein was seen in 52.1%, 66.2%, 72.6%, and 77.8%, respectively. DEL (MYC and BCL2 co-expression) accounted for 33.3% and did not occur more frequently in the non-GCB group (p=0.112). High proliferation index (MIB-1 antibody >90%) was detected in 26.2%. BCL6 protein expression was significantly more common among those having high proliferation activity compared to those having low proliferation activity (82.5% vs. 69.6%, respectively, with p=0.047). Based on the Hans algorithm, non-GCB and GCB types accounted for 63.7% (149/234 cases) and 36.3% (85/234 cases), respectively.

With FISH, *MYC* translocation was detected in 16 cases (7.3%), and all of them were positive for MYC protein expression. No case of *MYC* translocation was found with negative MYC protein expression. *MYC* translocation was statistically significantly associated with MYC protein expression (p<0.001) and male gender (p=0.049) but not with COO (p=0.086). There were only 4 cases (1.8%) of MYC GCN gain.

*BCL2* translocation was detected in 7.3%, all were in the GCB group (p<0.001). However, there was no significant difference in the prevalence of BCL2 protein expression by the presence of *BCL2* translocation (p=0.194). *BCL2* translocation seemed independent of gender (p=0.213). *BCL2* GCN gain was detected in 14.1% of cases, its presence was not significantly associated with BCL2 protein expression (p=0.068), COO groups (p=0.085), and gender (p=0.303).

*BCL6* gene rearrangement was confirmed in 21.4%, and it was significantly associated with COO (9 with GCB and 38 with non-GCB subtype; that is, 4.1% vs. 17.3%, respectively, with a p-value of 0.006). There was no significant association of *BCL6* gene rearrangement with BCL6 protein expression (p=0.315) or gender (p=0.693). There were only 2 cases with *BCL6* GCN gain.

There were 4 cases (1.8%) of dual *MYC* and *BCL2* translocations (2 cases of DEL, another two had only BCL2 protein overexpression), and all were in the GCB group.

*BCL2* gene alterations were more common with DEL compared to non-DEL (p=0.003), whereas the ratio of *MYC* gene alterations was similar between the groups (p=0.999).

### 3.2 Survival

At a median follow-up of 52 months (range: 0–131 months), 140 patients (56.7%) had disease progression or relapse. The overall response rate was 78.4% and the CR rate was 47.0%. The Kaplan-Meier estimate for EFS24 was 56.2% (CI: 50.4–62.8%). The 108 patients being not event-free at 24 months had a median OS of 7.1 months (CI: 6.5–8.0 months) after progression.

**Figure 1:**
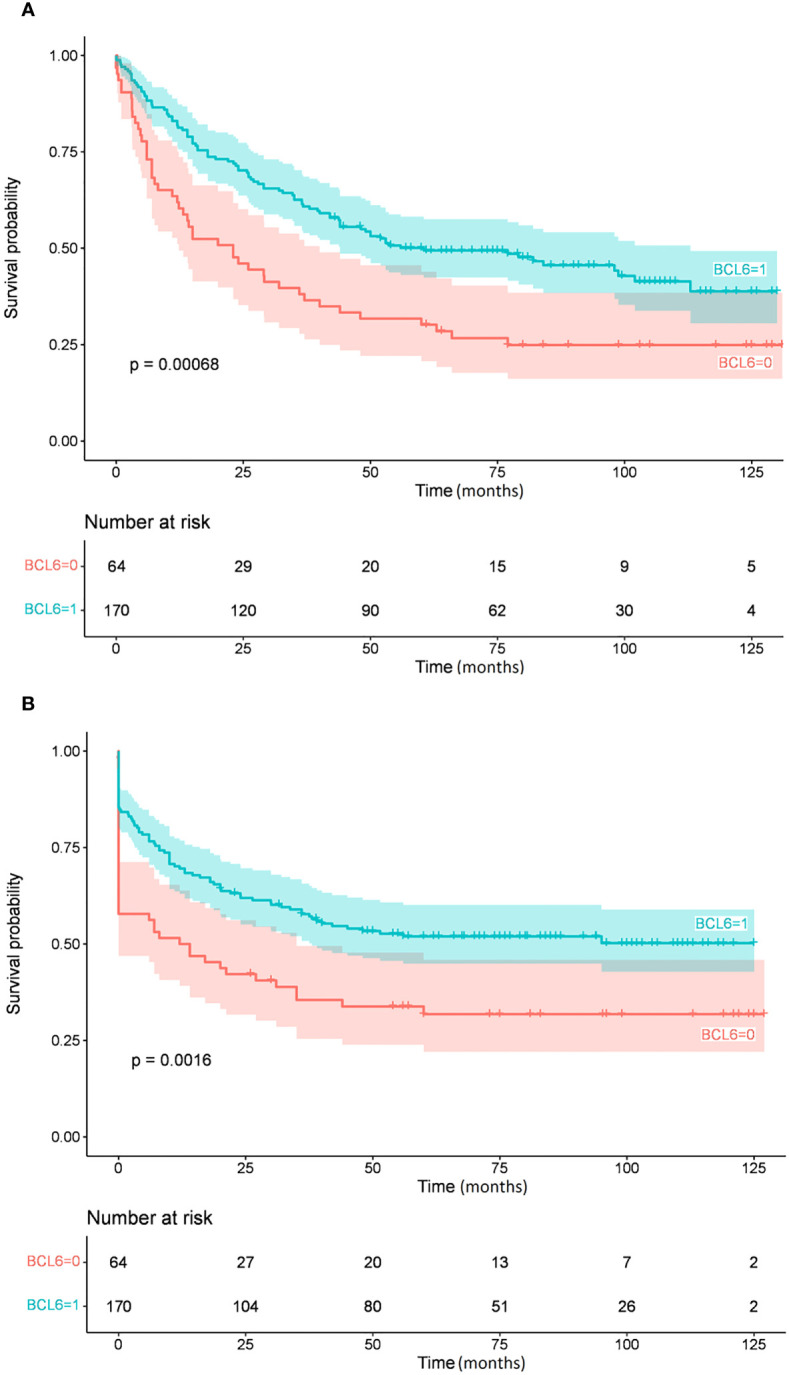
Overall survival **(A)** and event-free survival **(B)** by BCL6 protein expression.

**Figure 2:**
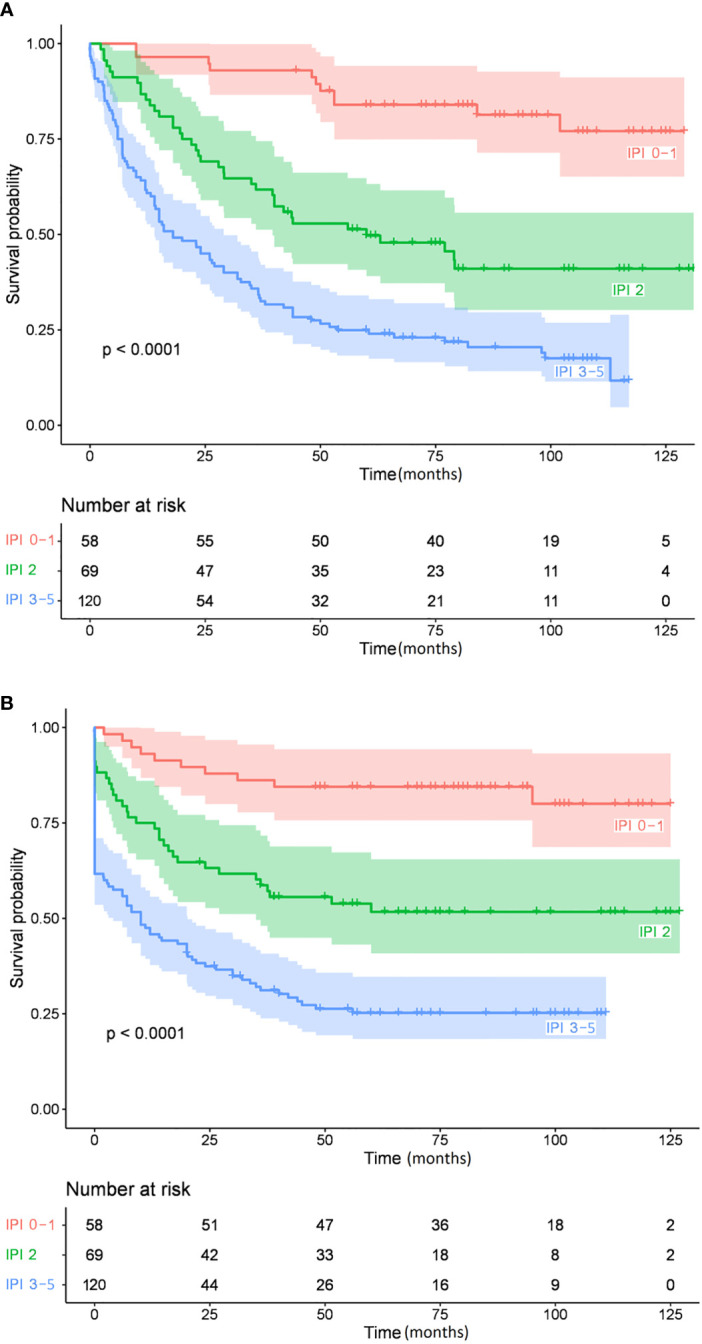
Overall survival **(A)** and event-free survival **(B)** by the International Prognostic Index.

**Figure 3:**
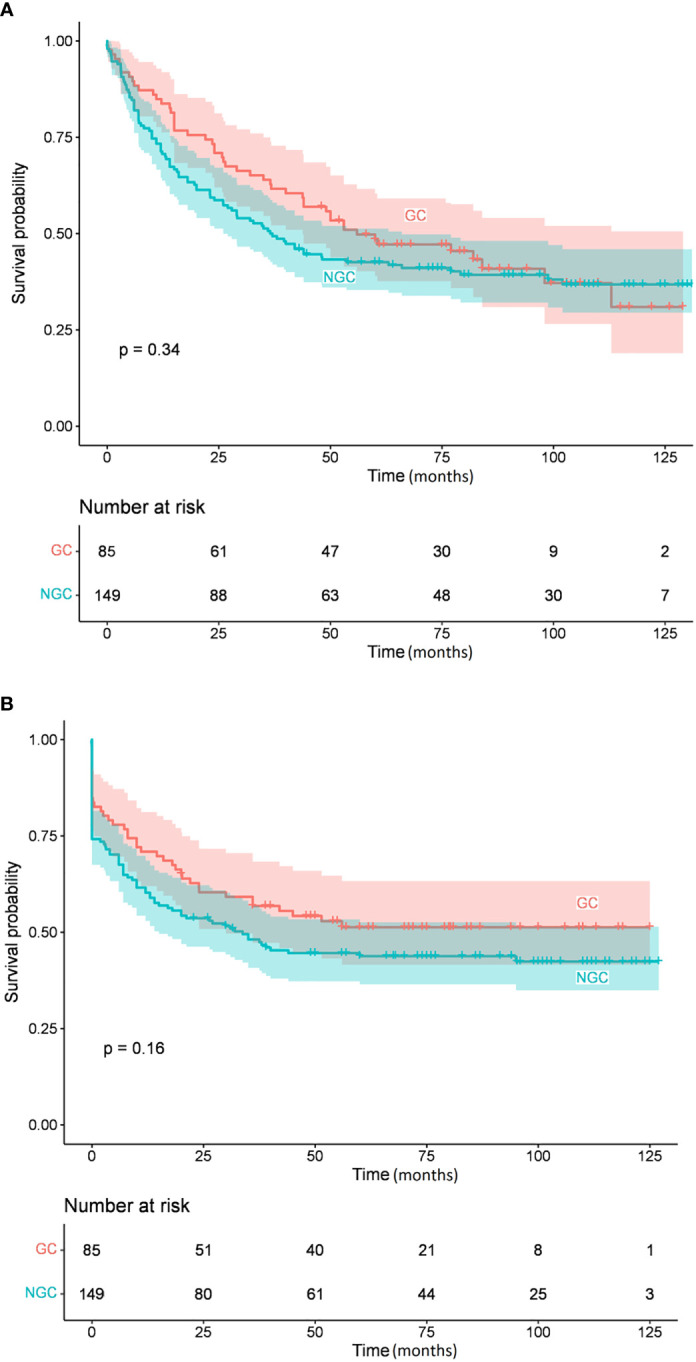
Overall survival **(A)** and event-free survival **(B)** by cell of origin subgroups.

**Table 4** summarizes the Kaplan-Meier survival estimates for all comparisons (234 and 220 patients had available IHC and FISH data, respectively). IPI and BCL6 expression were significant predictors of OS and EFS in univariate analysis (**Figures 1**, **2**, respectively), whereas MUM1 predicted only EFS. *BCL6* rearrangement, *BCL2* GCN gain, *IGH::MYC* translocation, and *IGH::BCL2* translocation did not have any prognostic impact on survival. Subgroup analysis by COO did not change the findings, nor did we find any difference in OS and EFS by COO subtype (**Figure 3**). Our results showed no difference in the 5-year survival in low-stage (I-II) and high-stage disease according to the COO. *BCL6* rearrangement did not predict OS and EFS in the non-GCB subgroup. DEL phenotype did not predict OS or EFS. We did not find any impact of double protein expression using MYC and BCL2 on the OS in low and high-stage diseases. The rate of double-hit lymphoma in this cohort of patients was 1.8% and the median OS for these group of patients was 33.8 months.

**Table 4:** Kaplan Meier estimates and log-rank tests for overall survival and event-free survival.

	Overall survival			Event-free survival		
	Median survival time (months)	95% CI	p-value for log rank test	Median survival time (months)	95% CI	p-value for log rank test
Gender						
Male	44	29.1**–**98.8	0.99	42	24**–**n.a.	0.65
Female	48	36.5**–**79		39	18.7**–**n.a.	
IPI						
IPI 0–1	n.a.	n.a.	<0.0001^*^	n.a.	n.a**–**n.a.	<0.0001^*^
IPI 2	60	39.6**–**n.a.		n.a.	35**–**n.a.	
IPI 3–5	18	14**–**29		10	4**–**20.2	
COO subtype						
GC	55.9	43.8**–**n.a.	0.34	n.a.	30**–**n.a.	0.16
NGC	36.8	26**–**66		33.7	15**–**n.a.	
CD10 expression						
Negative	39.8	32**–**77	0.49	37.5	20**–**n.a.	0.35
Positive	53	40**–**n.a.		n.a.	22**–**n.a.	
BCL6 expression						
Negative	23	12.3**–**40	0.0007^*^	13	0**–**35	0.002^*^
Positive	60.5	44**–**113		n.a.	38**–**n.a.	
MUM1 expression						
Negative	82	50**–**n.a.	0.054	n.a.	56**–**n.a.	0.009^*^
Positive	36.5	26**–**55.9		31	17**–**95	
Double expressor lymphoma						
Negative	48.1	36.5–84	0.4	44	26.3–n.a.	0.46
Positive	38.5	16–102		38	8– n.a	
MIB-1 index						
≤ 90%	40	29**–**60.5	0.071	30	16**–**95	0.072
> 90%	77	32.7**–**n.a.		n.a.	36**–**n.a.	
MYC expression						
Negative	44	32.7**–**77	0.8	37.5	21.2**–**n.a.	0.9
Positive	50	32**–**102		48	20**–**n.a.	
BCL2 expression						
Negative	60.5	32.7**–**n.a.	0.26	56	24**–**n.a.	0.46
Positive	42	32**–**63		38	20**–**n.a.	
*BCL6* translocation						
Negative	42.9	32**–**79	0.66	39	22**–**n.a.	0.37
Positive	29.1	15**–**n.a.		17	7**–**n.a.	
*BCL2* GCN gain						
Negative	44	35**–**79.1	0.97	39	27**–**n.a.	0.54
Positive	32	23.1**–**n.a.		18	9**–**n.a.	

CI, confidence interval; IPI, International Prognostic Index; COO, cell of origin; GC, germinal center B-cell like; NGC, non-germinal center B-cell like; GCN, gene copy number; n.a: median survival time not reached or data are insufficient for calculation. Asterisks indicate statistical significance.

**Table 5** summarizes the findings of the multivariate analysis of 220 patients. IPI score was a significant independent negative, whereas MIB-1 and BCL6 protein expressions were significant independent positive predictors of both OS and EFS.

**Table 5:** Multivariate Cox regression analysis for overall survival and event-free survival.

	Overall survival			Event-free survival		
	HR	CI	P-value	HR	CI	P-value
**Gender**	0.723	0.501**–**1.106	0.094	0.817	0.551**–**1.212	0.315
IPI						
IPI 1	ref.			ref.		
IPI 2	4.732	1.952**–**11.474	<0.001^*^	3.698	1.502**–**9.106	0.004^*^
IPI 3–5	10.451	4.515**–**24.193	<0.001^*^	8.600	3.702**–**19.976	<0.001^*^
**CD10 expression**	1.581	0.975**–**2.566	0.063	1.626	0.975**–**2.713	0.063
**BCL6 expression**	0.649	0.425**–**0.990	0.045^*^	0.623	0.398**–**0.976	0.039^*^
**MUM1 expression**	1.194	0.694**–**2.053	0.523	1.454	0.789**–**2.679	0.230
**MIB-1>90%**	0.581	0.364**–**0.927	0.023^*^	0.597	0.367**–**0.971	0.038^*^
**MYC expression**	1.071	0.724**–**1.585	0.732	1.141	0.758**–**1.719	0.528
**BCL2 expression**	0.993	0.656**–**1.503	0.973	0.952	0.6183**–**1.466	0.824
***BCL6* translocation**	0.967	0.617**–**1.514	0.883	1.057	0.667**–**1.676	0.813
***BCL2* GCN gain**	0.984	0.589**–**1.643	0.950	1.105	0.649**–**1.882	0.713

For molecular markers, the reference group is always the ‘negative’ group. Asterisks indicate statistical significance.

IPI, International Prognostic Index; ref, reference; GCN, gene copy number.

## 4 Discussion

The clinical and genetic heterogeneity of DLBCL still presents challenges in predicting response to treatment and prognosis. The 2016 revision of the World Health Organization classification of lymphoid neoplasms made it mandatory to classify the molecular subgroups of DLBCL into GCB and ABC subtypes (37) and this remains unchanged in the WHO 5^th^ edition published in 2023 (38). However, 10–15% of DLBCL cases cannot be categorized into one of the two COO groups and are termed unclassified DLBCL using gene expression studies. In the current study, we used IHC and FISH techniques on samples from DLBCL patients to classify rituximab-treated patients in many ways including by COO. Then, we interpreted survival data in this context.

In our cohort of patients, the COO phenotype failed to predict prognosis, which is surprising knowing that some studies have demonstrated significantly better survival for the GCB group (47, 48). However, the prognostic value of COO remains controversial, and other authors did not report any differences in overall prognosis based on COO, in line with our observations (49–51). **Table 6**. summarizes the COO distribution and survival data of DLBCL patients according to the immunophenotyping results reported in our study and other published reports. The controversy of the literature may be explained by many factors including but not restricted to the retrospective nature of the studies with a mixed pool of patients receiving various treatment regimens, in addition to a relatively short follow-up period (52).

**Table 6:** Distribution and survival data of DLBCL patients included in different studies according to immunophenotype.

Study group	n (%)	Survival data	P-value
**Our study**		**5-year OS (%)**	
GC	85 (36)	48.5	NS
NGC	149 (64)	42.6	
**Hans et al.** (8)		**5-year OS (%)**	
GC	64 (42)	76	<0.001^*^
NGC	88 (58)	34	
**Barrans et al.** (47)		**5-year OS (%)**	
GC	59 (35)	68	0.02^*^
NGC	110 (65)	58	
**Chang et al.** (48)		**Median OS (months)**	
GC	15 (39)	not reached	
Activated GC	12 (32)	15**–**26	<0.08^*^
NGC	11 (29)	38**–**40	
**Colomo et al.** (49)		**5-year OS (%)**	
CD10+GC	24 (21)	40	NS
CD10-GC	30 (26)	54	
NGC	60 (53)	42	
**Chadburn et al.** (51)		**1-year OS (%)**	
GC	33 (59)	70	NS
NGC	23 (41)	75	

GC, germinal center B-cell like; NGC, non- germinal center B-cell like; OS; overall survival; NS, not significant. Asterisks indicate statistical significance.

Factors that were independently associated with EFS in the multivariate analysis were IPI, high MIB-1 (>90%), and BCL6 expression. According to our results, BCL6 protein overexpression carries a positive prognostic effect on OS and EFS. BCL6 protein expression is considered as a hallmark of GC origin in DLBCL and it is associated with favorable outcome reported by some studies, consistent with our results (8, 19, 53–56). However, our findings demonstrated no significant association between BCL6 protein expression and *BCL6* translocation. In our study, the frequency of *BCL6* gene rearrangement was 21.4% and most of the cases were stratified into the non-GCB group according to the Hans algorithm. The association between the *BCL6* gene alteration and non-GCB phenotype was confirmed by other studies as well (17, 20, 57, 58). In our study, *BCL6* rearrangement had no prognostic impact on OS or EFS. Other studies yielded conflicting results showing that *BCL6* rearrangement was associated with a worse outcome (20, 59–63), reporting no significant associations at all (64–67), or implying an association with favorable outcomes (68, 69).

BCL2, MUM1, and MYC protein expressions did not emerge as independent prognostic variables in multivariate analysis. Unlike the high proliferation index (>90%) detected by MIB-1 antibody, which proved to be an independent predictor of good prognosis regarding OS and EFS. We did not find any significant difference in OS and EFS of DEL cases compared to the non-DEL cases.

Our finding suggesting no impact of BCL2 expression on the prognosis of patients with DLBCLis in line with the results of some previous publications (8, 70–73). However, other researchers have observed adverse outcomes with BCL2 protein overexpression (17, 26, 27, 74). In addition, in our study, there was no significant association between BCL2 protein expression and *BCL2* gene aberrations.

Finally, even in the rituximab era, there are still significant differences in OS and EFS across the IPI groups.

Our study has several strengths and limitations. The major strength of our work includes the size and coverage of the study population (247 DLBCL cases from 7 Hungarian centers), allowing a detailed and representative survival analysis. The major limitation of our work is the study’s retrospective design, which, as reflected by the number of excluded patients, resulted in lack of a full dataset in some analyses.

## 5 Conclusion

Successful integration of predictive and prognostic tools in DLBCL trials requires combination of clinical and molecular factors. Our study provides additional support to previously reported publications that DLBCL is characterized by heterogeneous molecular features and clinical outcomes. Based on our findings, only the IPI, BCL6 expression by IHC, and high (>90%) MIB-1 expression and not the other markers analyzed (CD10, BCL2, MUM1, MYC positivity, and gender) are independent predictors of OS and EFS in DLBCL. We did not find any difference in survival by GCB vs. non-GCB subtypes. These findings may improve prognostication in DLBCL and can contribute to designing further research in the area. However, considering the limitations of our study, these findings should be validated in prospective series.

## Data availability statement

The original contributions presented in the study are included in the article/supplementary material. Further inquiries can be directed to the corresponding author.

## Ethics statement

The studies involving humans were approved by The Committee of Science and Research Ethics (ETT-TUKEB) under reference number 50268-8/2017. The studies were conducted in accordance with the local legislation and institutional requirements. The participants provided their written informed consent to participate in this study.

## Author contributions

HA, IV-N, BA, and LP designed the research; NF and ZS analyzed data; HA, BA, and ZS drafted the manuscript; HA, LP, BA, IV-N, ZR, AG, BK, LK and ZS reviewed and approved the final version of the manuscript. All authors contributed to the article and approved the submitted version.
